# Intramuscular infiltration of a local anesthetic, lidocaine, does not result in adverse behavioural side effects in rainbow trout

**DOI:** 10.1038/s41598-018-28621-5

**Published:** 2018-07-06

**Authors:** F. Chatigny, C. M. Creighton, E. D. Stevens

**Affiliations:** 1 Department of Biomedical Sciences, Atlantic Veterinary College University of Prince Edward Island, Prince Edward Island Charlottetown, Canada; 2 Department of Companion Animals, Atlantic Veterinary College University of Prince Edward Island, Prince Edward Island Charlottetown, Canada

## Abstract

Fish are a useful animal model for research, but our improvement in some aspects of their welfare has not kept pace with their increased popularity for this use. For example, researchers rarely use analgesics. We evaluated the side effects of lidocaine, a local anesthetic widely used in human and veterinary medicine. We infiltrated lidocaine on each side of the dorsal fin (total 20 mg/kg) of young rainbow trout (15 fish per group) compared with infiltration with an equal volume of saline. We monitored behaviour of individual trout during the 4-hour trial. Food was presented 5 times during the trial (30 min, 1 h, 2 h, 3 h, 4 h after infiltration) and we analyzed behaviour for 1 minute before and after food presentation. Behaviour of Saline-Infiltrated trout compared with trout that received no infiltration showed that infiltration in and of itself had no statistically significant effects on trout behaviour. However, there were many statistically significant effects of Lidocaine-Infiltrated trout compared with Saline-Infiltrated trout; none of the side-effects were adverse.

## Introduction

Fish are increasingly popular animal models in research. According to the Canadian Council for Animal Care (CCAC) latest animal data report, fish are the largest group of all animals used in research in Canada^1^, with mice being the second. Currently the most commonly used fish are the zebrafish *Danio rerio*^2^ and rainbow trout Oncorhynchus mykiss (Walbaum 1792)^3^. When used for research, they may undergo procedures such as various routine surgical procedures including, but not limited to: fin biopsy, hypophysectomy, gonadectomy, implantation of telemetric devices, catheterization of the dorsal aorta and marking procedures such as tattooing^4–7^. Animal welfare regulations and guidelines have been created to ensure the wellbeing of fish used in research^8,9^, yet the use of analgesics in fish is limited by the lack of pharmacological information on the efficacy and side-effects of analgesic drugs on target fish species, as well as the ongoing debate as to whether or not fish “feel pain”^10^. This debate is not, however, directly linked to fish welfare as answering the pain question will not change our ability to improve fish wellbeing by treating the nocifensive responses that occur. A nocifensive response is a behaviour that may serve to protect the organism against injury, usually in response to a noxious stimulus. This type of response has been observed in fish and its existence is agreed upon by all parties in the debate^10,11^. Nocifensive responses often are associated with surgical procedures and veterinarians generally reduce them by providing appropriate perioperative care with a multimodal approach combining anesthetic and analgesic drugs^12–14^. One family of analgesic drugs, local anesthetics, is used to alleviate pain by interrupting nerve conduction in a specific region of the body, thus temporarily preventing the sensation of the noxious stimulus being conducted to the central nervous system. Local anesthetics interrupt nerve conduction by inhibiting the influx of sodium ions at voltage-gated sodium channels in axonal membranes. The mechanism involves binding of the drug to the H-gate or inactivation gate of the channel^15^. The appropriate use of local anesthetics can reduce the amount of anesthetic required as well as the overall requirements for systemic analgesia, in addition to providing sufficient localized desensitization for many minor surgical procedures^16^. Although it is often said that local anesthetics provide “analgesia”, they only produce anesthesia because they block all nerve conduction whereas analgesic agents block only nociceptive transmission. Local anesthetics tend to have a low cost and fast recovery period if used properly. However, local anesthetics may have adverse side effects that need to be considered when recommending their use. The myotoxic, cardiotoxic and neurotoxic effects of local anesthetics in mammals have been known since 1959^17^ and they are used experimentally to induce muscle degeneration. Also, some studies have reported that local anesthetics infiltrated in a wound area could delay wound healing. However, this is controversial because, as well summarized by Martin-Flores^18^, there are conflicting results in the literature. The most frequently used local anesthetics in veterinary medicine are the amide-linked drugs such as lidocaine hydrochloride (Xylocaine®)^16^.

There are mentions in the literature of local anesthetics being used for localized anesthesia in fish^19–26^, however, the efficacy, side-effects and safety of these agents has not been thoroughly investigated^27,28^. While we recognize and encourage the potential benefits of recommending the use of local anesthetics in fish, care must be taken not to cause harm with the use of the drugs themselves or by recommending ineffective drugs that would add unnecessary stressful procedures to the animals. This is why local anesthetics, just like any other drug, must be tested in fish before they can be properly used to treat them. The main objective of our project was to assess the potential side effects of local anesthetics in fish, by evaluating fish behaviour following lidocaine infiltration in rainbow trout. Infiltration differs from injection in that the needle is inserted fully and the solution is continuously infused as the needle is slowly withdrawn, whereas during injection the solution is infused at the injection site before the needle is withdrawn. Infiltration creates a path of solution the length of the needle.

## Results

There were no mortalities throughout the experiments. There were no statistically significant treatment effects when comparing the Non-Infiltrated group with the Saline-Infiltrated group (Table 1). Similarly, there were no metrics with statistically significant treatment*time interaction terms. The only statistically significant effect of time was for Differ (post-feed movement – pre-feed movement).

**Table 1:** Statistical tests comparing Non-Infiltrated (NI) with Saline Infiltrated (S) rainbow trout to test if infiltration in and of itself resulted in any change in any of the metrics.

Metric	Time	Treatment	Interaction
Recovery time	na	F _1,26_ = 0.99, p = 0.33	na
Ventilation rate	F_3,62_ = 2.20, p = 0.10	F _1,27_ = 0.29, p = 0.60	F_3,62_ = 0.6, p = 0.62
Jump	Z = 0.42, p = 0.67	Z = 0.16, p = 0.87	Z = 0.29, p = 0.77
Food consumption	X^2^ = 0.61, p = 0.43	X^2^ = 1.38, p = 0.24	X^2^ = 0.03, p = 0.86
Delay to eat		Z = 0.29, p = 0.78	
Pre-feed move (l/m)		Z = −0.30, p = 0.76	
Post-feed move (l/m)		Z = 0.82, p = 0.42	
Differ = Post – Pre (l/m)	**F**_**4**,**99**_** = 2**.**72**, **p = 0**.**034**	F _1,52_ = 1.54, p = 0.22	F_4,99_ = 0.67, p = 0.61
Post-feed move (% of total)	F_4,71_ = 1.58, p = 0.19	F _1,28_ = 0.37, p = 0.55	F_4,71_ = 0.53, p = 0.71

The F values are from ANOVAs; the X^2^ and Z values for Jump and Food consumption are from logistic regressions; Z values for Delay to eat and raw movement data are from non-parametric tests (Wilcoxon); n = 15 per treatment group.

On the other hand, there were many statistically significant treatment effects when comparing the Saline-Infiltrated group with the Lidocaine-Infiltrated group; 7 out of 9 metrics differed significantly (Table 2). There were no metrics with statistically significant treatment*time interaction terms. The only statistically significant of effect of time was for Differ (post-feed – pre-feed movement).

**Table 2:** Statistical tests comparing Saline Infiltrated (S) with Lidocaine Infiltrated (L) rainbow trout to test effect of lidocaine.

Metric	Time	Treatment	Interaction
Recovery time	na	F_1,26_ = 0.75, p = 0.39	na
Ventilation rate	F_3,58_ = 1.15, p = 0.34	F_1,27_ = 7.79, **p = 0**.**01**	F_3,58_ = 0.77, p = 0.52
Jump	X^2 = ^0.69, p = 0.41	X^2 = ^3.90, **p = 0**.**048**	X^2 = ^2.91, p = 0.09
Food consumption	Z = −0.19, p = 0.85	Z = 2.10, **p = 0**.**036**	Z = −0.17, p = 0.86
Delay to eat		Z = 2.81, **p = 0**.**006**	
Pre-feed move (l/m)		Z = 0.03, p = 0.97	
Post-feed move(l/m)		**Z = −3**.**06**, **p = 0**.**003**	
Differ = Post – Pre (l/m)	**F**_**4**,**100**_** = 2**.**64**, **p = 0**.**038**	F_1,48_ = 6.28, **p = 0**.**016**	F_4,100_ = 0.68, p = 0.61
Post-feed move (%)	F_4,98_ = 0.87, p = 0.48	F_1,56_ = 6.40, **p = 0**.**014**	F_4,98_ = 0.87, p = 0.49

The F values are from ANOVAs; the X^2^ and Z values for Jump and Food consumption are from logistic regressions; Z values for Delay to eat and raw movement data are from are from non-parametric tests (Wilcoxon); n = 15 per treatment group.

**Figure 1:**
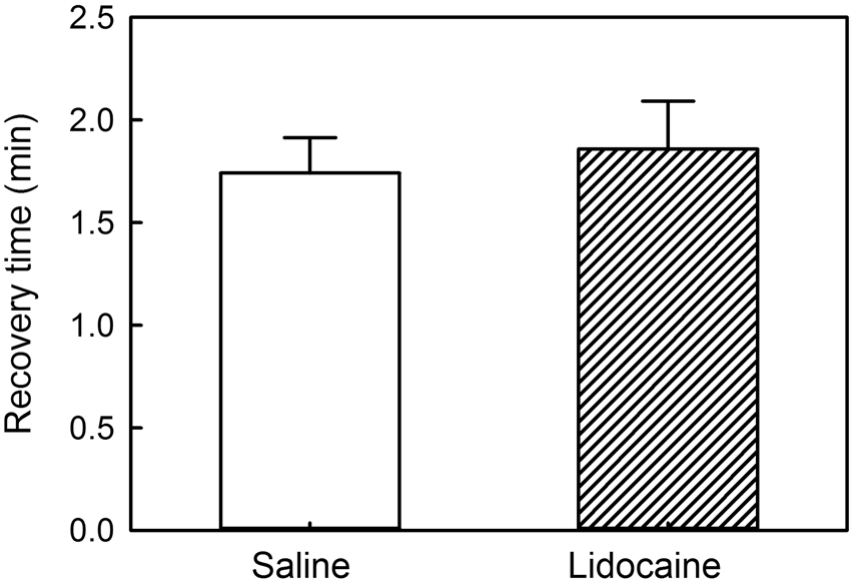
Lidocaine infiltration did not prolong recovery time in rainbow trout after anesthesia. Recovery time is time to regain an upright swimming attitude after MS222 (tricaine MS). Means ± 95% CI, n = 14/treatment group.

**Figure 2:**
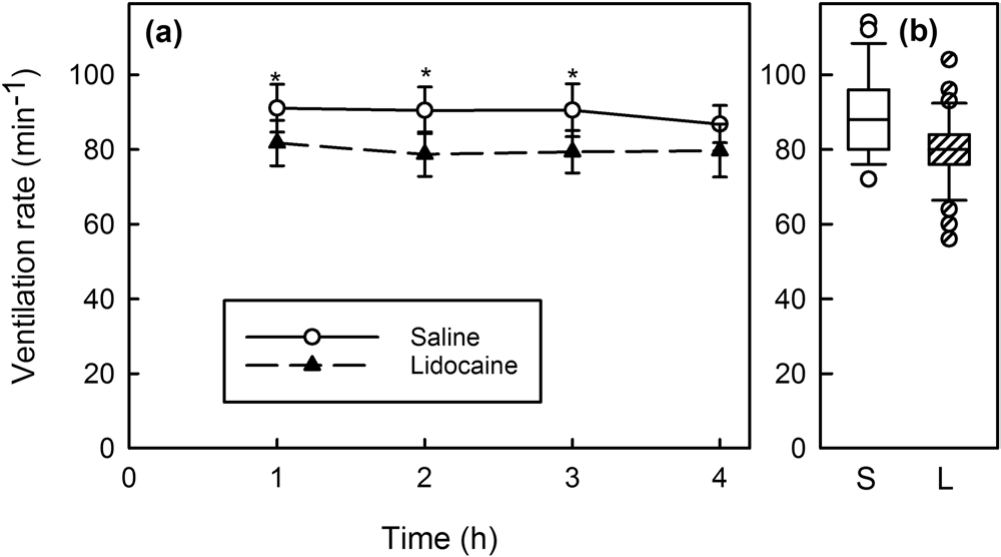
Ventilation rate was lower in Lidocaine-Infiltrated trout at 1, 2, and 3 hours after infiltration (*). (**a**) Means ± 95% CI, n = 14 or 15/treatment group. (**b**) Boxplots of data over all 4 time periods.

**Figure 3:**
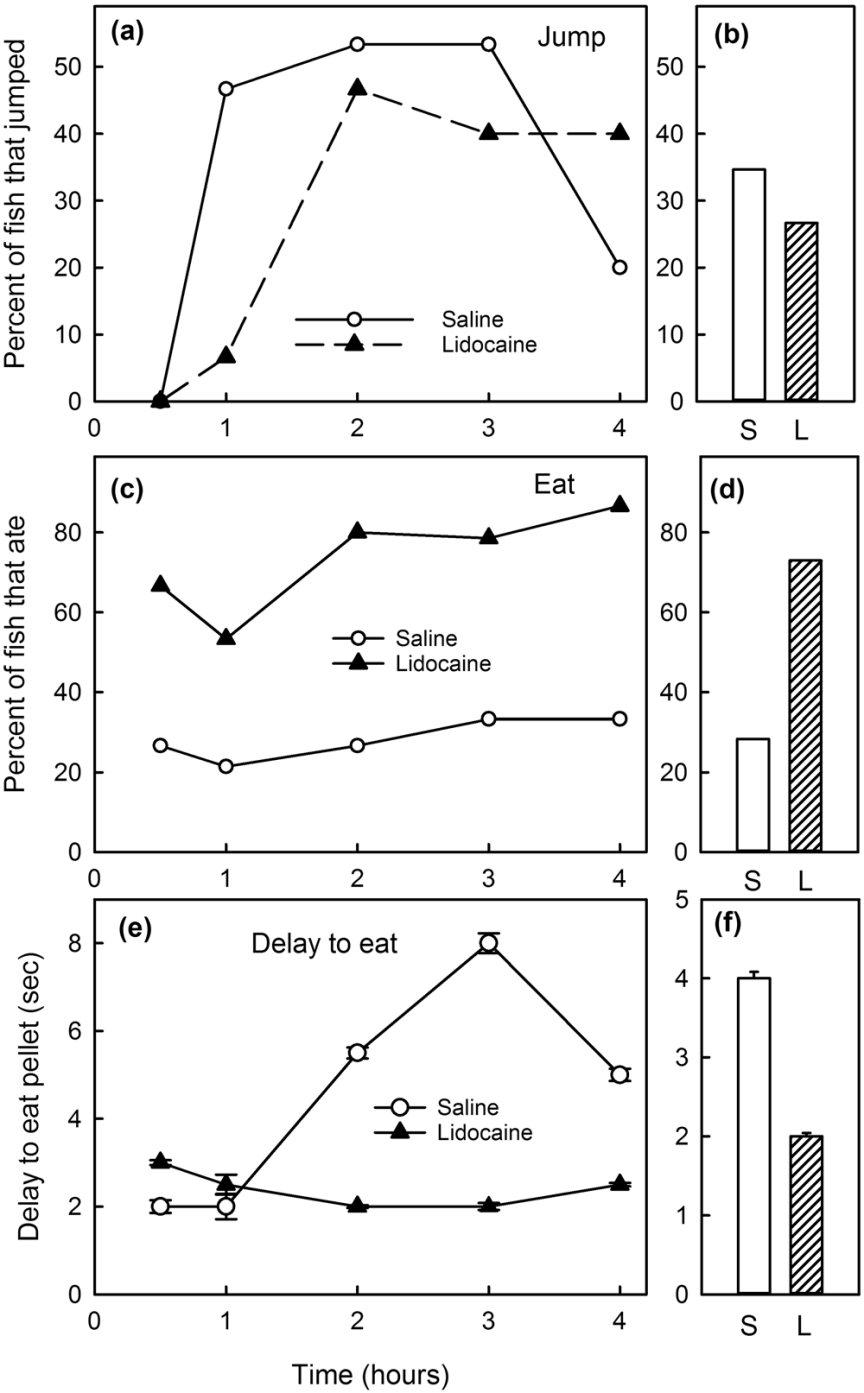
The proportion of rainbow trout that exhibited escape behaviour was less in Lidocaine-Infiltrated trout, especially 1 hour after infiltration. (**a**) Percent of trout that jumped at each time point, (**b**) percent over all time periods after infiltration. The proportion that consumed a pellet when food pellets were presented was greater in Lidocaine-Infiltrated rainbow trout at all time points (**c**) and over all time periods (**d**) after infiltration. When a trout did eat a pellet, the delay from the time it was presented till they consumed it was less in Lidocaine-Infiltrated trout at 2, 3, and 4 hours after infiltration (**e**) and over all time periods (**f**); medians ± 95%CI, n = 15/treatment group.

Recovery time, the time to regain equilibrium after anesthesia, was not significantly altered by the lidocaine infiltration and averaged 1 min 48 sec (Table 2, Fig. 1). Ventilation rate, based on opercular movements, was about 10% less in Lidocaine-Infiltrated trout compared with Saline-Infiltrated trout (Fig. 2) and this difference was statistically significant at 1, 2, and 3 hours after treatment, but not at 4 hours after treatment (Table 2, Fig. 2). Escape behaviour, based on a fish jumping completely out of the water, was about 23% less in Lidocaine-Infiltrated trout compared with Saline-Infiltrated trout (Fig. 3A,B) and this difference was statistically significant (Table 2, Fig. 3A). No trout jumped at 30 min post-treatment time point, but almost 40% jumped 1 to 4 hours post-treatment. The largest difference between groups occurred 1 hour post-treatment and was statistically significant (Fisher’s exact test; p = 0.035). Almost three times as many fish in the Lidocaine-Infiltrated group consumed food pellets when they dropped, compared with the Saline-Infiltrated group and this difference was statistically significant (Table 2, Fig. 3C,D). When compared at each time point, this difference was statistically significant at all time points except 1 hour post-treatment. When the trout did eat, the Lidocaine-Infiltrated trout tended to take the pellet more quickly than the Saline-Infiltrated trout (Fig. 3E,F); the overall difference was statistically significant (Table 2). Because so many more trout in the Lidocaine-Infiltrated group consumed food pellets when they dropped, statistical comparisons at different time points are not meaningful.

**Figure 4:**
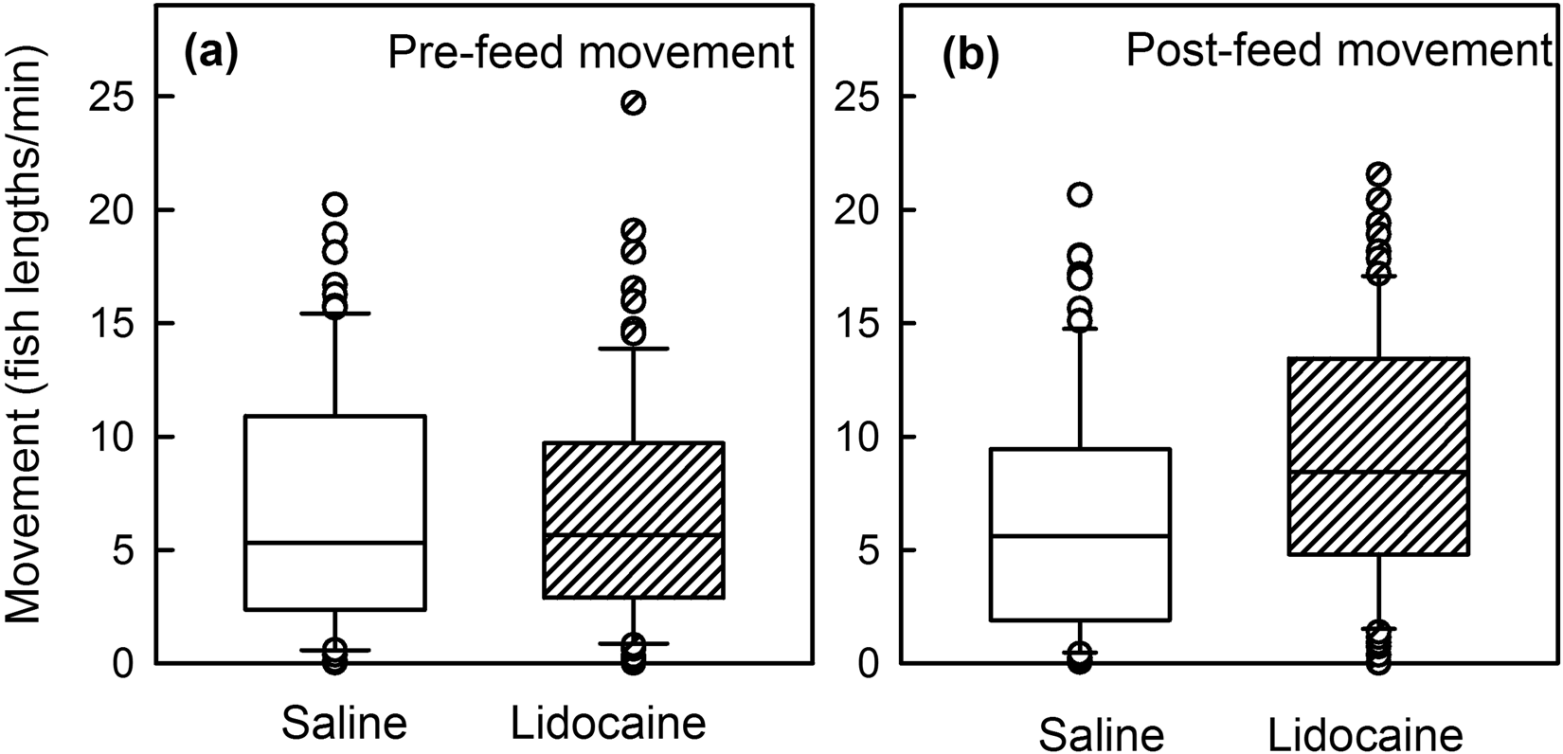
Raw movement data. Boxplots of raw movement data illustrate the non-normal nature of the distributions and that movement was greater in the Lidocaine-Infiltrated trout after food presentation but not before food presentation (Wilcoxon).

**Figure 5:**
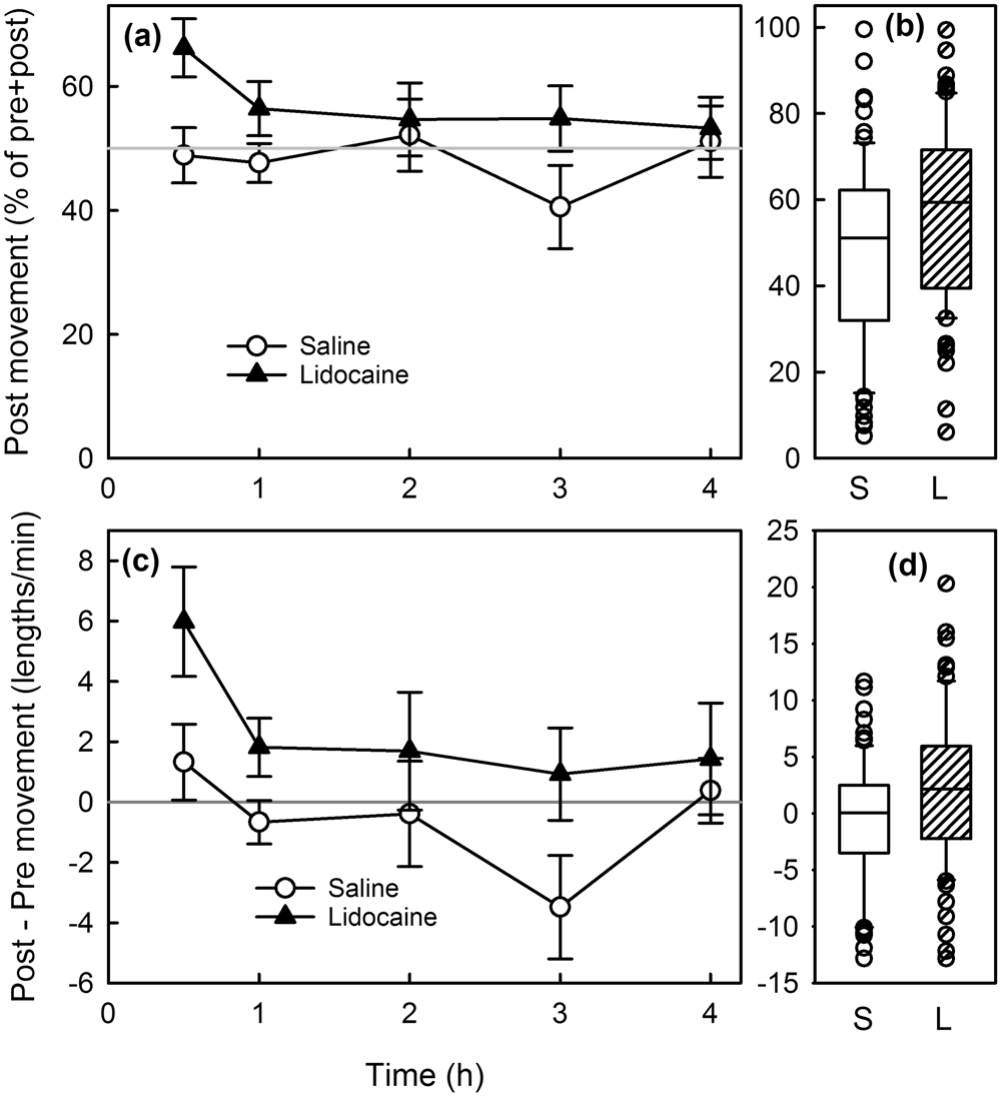
Post-feed movement was greater for Lidocaine-infiltrated trout. Post-feed movement as a % of total (**a**) (i.e., 100% * Post-feed movement/(pre-feed movement + post-feed movement) and differ (**b**) (post-feed movement – pre-feed movement) values were greater for Lidocaine-Infiltrated trout compared with Saline-Infiltrated trout at all time periods; the differences were statistically significant at 0.5 h and at 3 h after infiltration.

Statistical comparisons on the non-normally distributed raw movement data indicated that for movement the treatment effect was significant after feeding but not before, and that Lidocaine-Infiltrated trout tended to move more than the Saline-Infiltrated trout (Table 2, Fig. 4a,b). Normalized movement data calculated as Post% (post/(pre + post)) and Differ (Post − Pre) supported that result (Table 2, Fig. 5a–d). These two metrics are moderately correlated (R-Sqr = 0.75). For both metrics (Post% and Differ), the difference was statistically significant at 0.5 and 3 hours post-treatment but not at the other time points.

## Discussion

Before recommending that drugs be used in any animal, it is important to have an informed idea of their potential side effects. The goals of the present experiments were first, to estimate side effects of the infiltration procedure in and of itself, and second, to estimate the behavioural effects of lidocaine infiltration when used as a local anesthetic compared with saline infiltration in trout.

Infiltrationalone had no noteworthy side effects on the behaviour of rainbow trout. However, when comparing infiltration of lidocaine with saline, there were some significant effects. In particular, trout infiltrated with lidocaine had a lower ventilation rate, exhibited less escape behaviour, were more inclined to eat when food was presented, and moved more after food presentation relative to Saline-Infiltrated controls. In the present experiments lidocaine was not blocking the effect of a noxious stimulus because none was applied.

We tested the effect of lidocaine on the time to recover from TMS (Tricaine Methanesulfonate, or MS222) anesthesia because lidocaine added to ambient water has been used as an anesthetic in fish^29–35^, and we hypothesized that the two drugs (TMS and lidocaine) might act synergistically and lengthen recovery time. Our results showed that even though we infiltrated a relatively large dose of lidocaine, time to recover from TMS anesthesia did not differ between trout infiltrated with saline and those infiltrated with lidocaine.

We did not anticipate that the trout would try to escape from the test arena and only added this variable after looking at the videos. There are many possible explanations for our observation that Lidocaine-Infiltrated trout had a lower ventilation rate, exhibited less escape behaviour, were more inclined to eat when food was presented, and moved more after food presentation. Lidocaine gradually diffusing from the infiltration site to the systemic circulation could have a central sedation effect or anxiolytic effect. The absence of a difference in recovery time from TMS anesthesia argues against lidocaine having a central sedation effect. Admittedly, this is a weak argument because TMS is rapidly removed from the circulation and intramuscular lidocaine is slow to reach the central circulation; in mammals it takes 20–30 minutes to reach peak values. On the other hand, injection of lidocaine into the medial prefrontal cortex of the rat brain does result in anxiolytic-like effects^36^. Thus, it is possible that diffusion of lidocaine from the infiltration site to the central circulation had an anxiolytic effect on the trout brain. In mammals, lidocaine infiltration does not reduce the cortisol response during a stressful surgery^37,38^ but insofar as we know, there are no similar experiments in fishes.

The ventilation frequency was lower in Lidocaine-Infiltrated trout and closer to values often reported as being “normal” in rainbow trout than in Saline-Infiltrated trout. Insofar as we know, there are no measurements of ventilation frequency telemetered from rainbow trout in the wild; the mean rate was 46 per min for a single brown trout (*Salmo trutta*) during periods of no movement in a lake at 11 °C^39^. Control values for ventilation frequency of rainbow trout in laboratory situations range from 46–95 per min. The low rate (46) is from large trout (743 g) at 10 °C that were fasted for 1 to 2 weeks prior to as well as during the measurements, in the dark, and during periods of no movement^40^. Control or pre-treatment values for individual rainbow trout in studies from Sneddon’s laboratory are remarkably consistent at 52 per min trout^26,41–45^; Hargis reported a value of 58 per min^46^. Other values for rainbow trout kept individually but cannulated or with electrodes typically report higher values: 70 per min^47^, 77^48^, 95^49^. Many factors including fish size, strain (i.e., genetic background), social status and water temperature likely influence the ventilation rate. It is unlikely that the higher ventilation frequency in the Saline-Infiltrated trout and Non-Infiltrated trout in our study is due to being isolated in the test arena because in all other studies referenced above, the trout were isolated.

All three groups were similar in that very few fish exhibited escape behaviours at the first time point, 30 minutes after recovery from TMS anesthesia; this is likely an effect of the TMS anesthesia. This result supports the often-adopted practice of waiting half an hour before initiating observations for behavioural experiments, although some relevant information may be lost by ignoring this first half hour^50^.

The most likely explanation for our observation that Lidocaine-Infiltrated trout moved more after food presentation than Saline-Infiltrated trout is that the greater activity was associated with chasing and eating food pellets circulated by the water pump in the test arena. However,this explanation is not very satisfying because we do not have a good explanation why Lidocaine-Infiltrated trout were more inclined to feed.

All the above effects could be explained by the fact that the infiltration itself is an additional stressor that the lidocaine effectively blocks. This effect is probably local, as no other signs of general effects were seen, but a central anxiolytic effect cannot be dismissed.

While no adverse side effects were observed during the experiments outlined here, some were observed during later experiments by our laboratory. The first instance of observed side effects happened as an incidental finding. Two fish were mistakenly infiltrated with non-diluted lidocaine corresponding to a dose of nearly 180 mg/kg. One fish died within an hour of the infiltration. The other fish swam ventral side up for an hour and then was euthanized as per the approved protocol. Two fish infiltrated with an approximate dose of 150 mg/kg survived; one swam normally while the other showed loss of equilibrium for about an hour and then recovered. Respiration appeared normal in both fish. The lethal dose in humans is about 20 mg/kg^51^; 75 mg/kg is lethal in dogs^52^ and the LD50 in mice is 24 mg/kg^53^. Thus, rainbow trout appear to be able to tolerate much higher doses of lidocaine than mammals, possibly due to their lower body temperature and lower metabolic rate.

There are some potential caveats of our experiment. First, a fish was considered to have fed only if it did so 1 minute following food presentation. While most fish seemed to feed fairly quickly after food presentation, some fish could have fed later. Second, the 4 h duration of the experiment was based on the usual duration of effect of lidocaine in mammals^54^. The side effects that we observed occurred fairly quickly after the infiltration, thus it is possible, but unlikely, that we missed later side effects. Fishes’ physiology is different from mammals’; thus, it is likely that the onset and duration of lidocaine’s effect might differ. In as much as the purpose of lidocaine infiltration is to improve welfare during surgery, we are most interested in relatively short-term effects. Third, movement was assessed as distance moved; subtle elements of fish swimming were likely missed. For example, most fish made limited movements until food was presented and then they would either dart a few times to catch the food pellets or swim all over the test arena. Distance moved does not distinguish between these different types of movement, but these differences are captured to some extent in other variables (fed, delay to feeding, escape behaviour).

Mammals are usually the point of reference because local anesthetics have not been studied extensively in other groups of animals^27,28^. Insofar as we know there are only two experimental studies using local anesthetics for local anesthesia in fish. Chervova^19^ used a different local anesthetic, Novocaine® (procaine), and reported that injection of the drug “fully blocked up nociceptive responses”. Side effects were not monitored because the fish were restrained during the trials. Additionally, details regarding how the efficacy of procaine was assessed or timing of the tests relative to its administration are not reported and, no specific dose is given. Mettam *et al*. (2011) attempted to assess the efficacy of 2 doses of lidocaine, 9 and 18 mg/kg, and recommend using a dose of 9 mg/kg^26^. Their recommendation does not appear to be supported by their results insofar as none of their metrics differed between fish receiving the noxious stimulus and treated with lidocaine (9 mg/kg) compared with those receiving the noxious stimulus and a dose of zero. Our experiment was similar to that of Mettam *et al*.: both used rainbow trout, one of the doses they used, 18 mg/kg, was similar to our dose of 20 mg/kg, and both evaluated ventilation rate, movement and delay to food consumption. The studies differ in that they injected into the lips whereas we infiltrated into epaxial muscle adjacent to the dorsal fin, they did not evaluate parameters in the first 30 min or later than 3 hours, and their sample size was very small (n = 5) relative to ours (n = 15).

There were no adverse side effects of lidocaine infiltration on behavioural metrics even though the dose we used was higher than that normally used in mammals. The dose of 20 mg/kg showed some potentially beneficial effects as fish infiltrated with lidocaine were more likely to feed and had a lower ventilation rate. Doses at least up to 20 mg/kg appear safe in rainbow trout with respect to behavioural metrics. However, in a separate experiment, infiltration of a dose of 10 mg/kg of lidocaine into the same site on one side of the dorsal fin resulted in hemorrhage, inflammation and muscle degeneration and necrosis of skeletal muscle and connective tissues^55^. These lesions were either nearly or completely absent by day 30. Our results do not allow us to recommend an ideal dose of lidocaine for infiltration, as this was not the goal of this experiment. Further studies are needed to confirm an effective dose for local anesthetics used as local infiltration for most fish species.

## Material and Methods

### Animals and housing

Forty-five rainbow trout (Oncorhynchus mykiss) from Ocean Trout Farms, Brookvale, PE arrived May 11, 2016 and were maintained at the aquatic facility of the Atlantic Veterinary College (AVC) at the University of Prince Edward Island (UPEI). They were held in 200-L tanks. Mean fish weights during the experiment were 125.2 ± 32.3 grams, while mean lengths were 22.6 ± 2.5 cm (mean ± SD). The water temperature was 11 ± 1 °C, photoperiod was 12:12 and they were fed pellet food daily 1% / body weight per day each morning (EWOS® Transfer 3 mm diet for salmonids). Fish were acclimated to holding conditions for at least a week before the experimental trials. They were maintained and treated according to the ethical guidelines of the CCAC; this project was approved by the UPEI Animal Care Committee (Protocol Number: 15–019–6006255).

Fish were anesthetized individually in 1.5 L of water containing TMS (80 mg/L, Aqua Life TMS (MS 222), Tricaine Methanesulfonate, Syndel Canada) buffered with equal amounts of sodium bicarbonate (Sigma-Aldrich, St. Louis, MO). Two elliptical test arenas (52 × 27, water depth 11 cm) were placed side-by-side on a large water table. Each contained an air stone and a small pump to create a slow water flow and was illuminated with light reflected off a white surface placed 2 m above the arenas.

### Procedures

Fish were transferred to the trial arenas between 16–18 hours so they could acclimate to the trial arena overnight and be fasted for a minimum of 24 h. Fish were anesthetized at 12 h, transferred to the surgical table and treated. There were 3 treatment groups: Non-Infiltrated fish were anesthetized, placed on the surgical table for 1 min, then returned to their test arena (n = 15); Saline-Infiltrated fish were anesthetized, placed on the surgical table, infiltrated with 0.25 mL of sterile saline on each side of their dorsal fin, then returned to their test arena (n = 15); Lidocaine-Infiltrated fish were anesthetized, placed on the surgical table, infiltrated with 0.25 mL of lidocaine on each side of their dorsal fin, then returned to their test arena (n = 15). The dose of lidocaine was 20 mg/kg with one-half infiltrated on either side of the dorsal fin and was calculated based on the average weight of the fish. Saline was sterile 0.9% Sodium Chloride for Injection USP (Hospira Healthcare Corporation, Montreal, QC). Lidocaine was lidocaine hydrochloride 2% (CAS 6108-05-0; Lurocaine, Vetoquinol, Lavaltrie, QC) diluted in sterile saline (1 lidocaine:4 saline for a final concentration of 5 mg/mL). No adjuvants were added. For infiltrations, a 25 G syringe needle on a 1 mL syringe was inserted 5 mm behind the caudal end of the dorsal fin and then fully inserted cranially. The solution was then incrementally infiltrated as the needle was slowly withdrawn caudally. The infiltration volume was kept constant at 0.25 mL per site for a total of 0.5 mL per fish. The doses of lidocaine were calculated based on the average fish weight; each fish was given the same volume of drug so as to mimic the conditions in the field where an approximate weight is used^56^.

After treatment, the fish were placed back in their corresponding arena and allowed to recover from anesthesia (i.e., regain a normal vertical position and able to swim upright for 10 consecutive seconds). Fish were fed using automatic feeders (Everyday Fish Feeder, Eheim) at 5 time points (0.5 h, 1 h, 2 h, 3 h, 4 h) and we analyzed their activity 1 minute before and after each feeding. Trials were started as soon as the fish recovered (time = zero) and the experimenter left the test room. After the 4 h test period the fish were anesthetized again to obtain weight and fork length.

Recovery time was recorded and calculated as the time between a fish’s return to the arena and the moment it regained a vertical position and was able to swim upright for 10 consecutive seconds. Fish behaviour was recorded continuously during the 4 h trial with a camcorder (JVC AVCHD EVERIO, model GZ-VX700BU) placed directly above the arenas and intermittently at each feeding time point using the remote-control function of a GoPro (GoPro Hero3 White) placed on the side of each arena. Fish’s activity was analyzed using LoliTrack® (ver. 4.1, Loligo Systems, Tjele, Denmark). Raw distance moved over time was filtered with a 7-point median filter to remove glitches (Microsoft Excel®). Total movement for 1 minute before food presentation (pre-feed movement) and 1 minute after food presentation (post-feed movement) were divided by the fork length; movement is reported as lengths/min. These movement values were used to estimate 2 more parameters: the amount of movement after each food presentation (Post%) relative to the total movement (Post% = post-feed-movement/(pre-feed movement + post-feed movement)), and the difference between pre- and post-feed movement (Differ = post-feed movement – pre-feed movement). Fish were recorded as having fed only if they ingested pellets in 1 minute after food presentation at each time point. If food pellets were consumed, the delay between the pellets dropping to consumption was recorded. Ventilation rate was estimated by counting the number of opercular movements per minute. Escape behaviours were recorded by assessing whether or not the fish attempted to jump out of the test arena. This behaviour was characterized by the fish jumping out of the water completely, usually at the edge of the arena, but the fish was unable to jump out of the arena because of its high walls and fell back into the water.

### Statistics

SAS 9.2 (SAS Inst Inc., Cary, NC, USA) was used for all statistical tests except for the test for normality (Ryan-Joiner) and the test for homogeneity of variance (Levene); Minitab® (17.3) was used for these two tests. The level of significance for all tests was two-tailed and set at p < 0.05. Post-hoc tests were done using contrasts (slice option in SAS). Recovery time was tested with a one-way (ANOVA) (SAS proc glm) after deleting 2 outliers (more than 3 standard deviations away from the mean) and a Box-Cox transformation when comparing Non-Infiltrated with Saline-Infiltrated; and tested after deleting 2 outliers but no transformation was needed when comparing Saline-Infiltrated with Lidocaine-Infiltrated. Ventilation rate was tested using a repeated measures two-way ANOVA (SAS proc mixed) after deleting one extreme outlier and a Box-Cox transformation when comparing Non-Infiltrated with Saline-Infiltrated; and after a Box-Cox transformation when comparing Saline-Infiltrated with Lidocaine-Infiltrated. Jump and food consumption were tested with a repeated measures binary logistical regression (SAS proc genmod). No fish jumped during the first time period (30 min post-treatment); that point was excluded in the statistical analysis. Delay to feed exhibited a very non-normal distribution that could not be transformed to a normal distribution with any Box-Cox transform (SAS proc transreg) and therefore was tested non-parametrically (Wilcoxon) to compare distributions (SAS proc npar1way).

Raw data for movement (pre-feed movement, post-feed movement, or their sum) all exhibited very non-normal distributions that could not be transformed to a normal distribution with any Box-Cox transform (SAS proc transreg). The raw movement was tested non-parametrically (Wilcoxon) to compare distributions (SAS proc npar1way) and although it is possible to test this data assuming a negative binomial distribution rather than a normal distribution no additional statistical inference was achieved. Differ (post-feed movement – pre-feed movement) and Post% (post-feed movement as a percent of the sum of pre-feed movement + post-feed movement) were tested using a repeated measures two-way ANOVA (SAS proc mixed).

### Data availability

The datasets generated during and analysed during the current study are available in the repository [https://data.upei.ca], at 10.11571/upei-roblib-data/researchdata:501.
